# Postoperative radiotherapy to stabilize a tumor embolus in clear cell renal cell carcinoma: A case report

**DOI:** 10.3892/ol.2014.2421

**Published:** 2014-08-05

**Authors:** ZHI-HUA GONG, LV-JUN YAN, JIAN-GUO SUN

**Affiliations:** Department of Oncology, Cancer Institute of People’s Liberation Army, Xinqiao Hospital, Third Military Medical University, Chongqing 400037, P.R. China

**Keywords:** intensity modulated radiation therapy, clear cell renal cell carcinoma, tricuspid inflow obstruction

## Abstract

Superior vena cava (SVC) syndrome results from clear cell renal cell carcinoma and is a challenge in clinical practice due to its pathological complexity and a lack of research data. The current study presents a 49-year-old female with symptoms of exertional dyspnea and increased fatigue, which had persisted for 15 months, as well as bilateral edema in the lower limbs for two days. A transesophageal echocardiogram demonstrated a right atrial mass originating from the inferior vena cava (IVC; size, 14×8 cm) that caused a tricuspid inflow obstruction. Following a partial resection of the thrombus, a clear cell renal cell carcinoma was identified by histological examination. The patient received intensity-modulated radiation therapy following refusal of other therapeutic methods. The eleven-month follow-up indicated that the tumor on the kidney and IVC was stable. Intensity-modulated radiation therapy may be beneficial to patients with clear cell renal cell carcinoma and SVC syndrome. However, additional studies are required to obtain further data regarding the treatment of this syndrome.

## Introduction

Diagnosis and treatment of superior vena cava (SVC) syndrome, resulting from clear cell renal cell carcinoma, is a challenge in clinical practice. SVC syndrome is a complication caused by obstruction of the SVC (either compressed or internally obstructed) and/or compression of the heart. Management of the SVC syndrome induced by malignant conditions is based on anticancer therapy and the relief of symptoms (1). Renal carcinoma has a high frequency of renal vein and inferior vena cava extension. The efficacy and safety of treatments for renal carcinoma with SVC involvement requires clarification in future studies. The present case study describes the case of a patient who presented with a large, malignant thrombosis of the SVC and received intensity-modulated radiation therapy following an intra-atrial tumor thrombus resection, which resulted in stable local control.

## Case report

**Figure 1:**
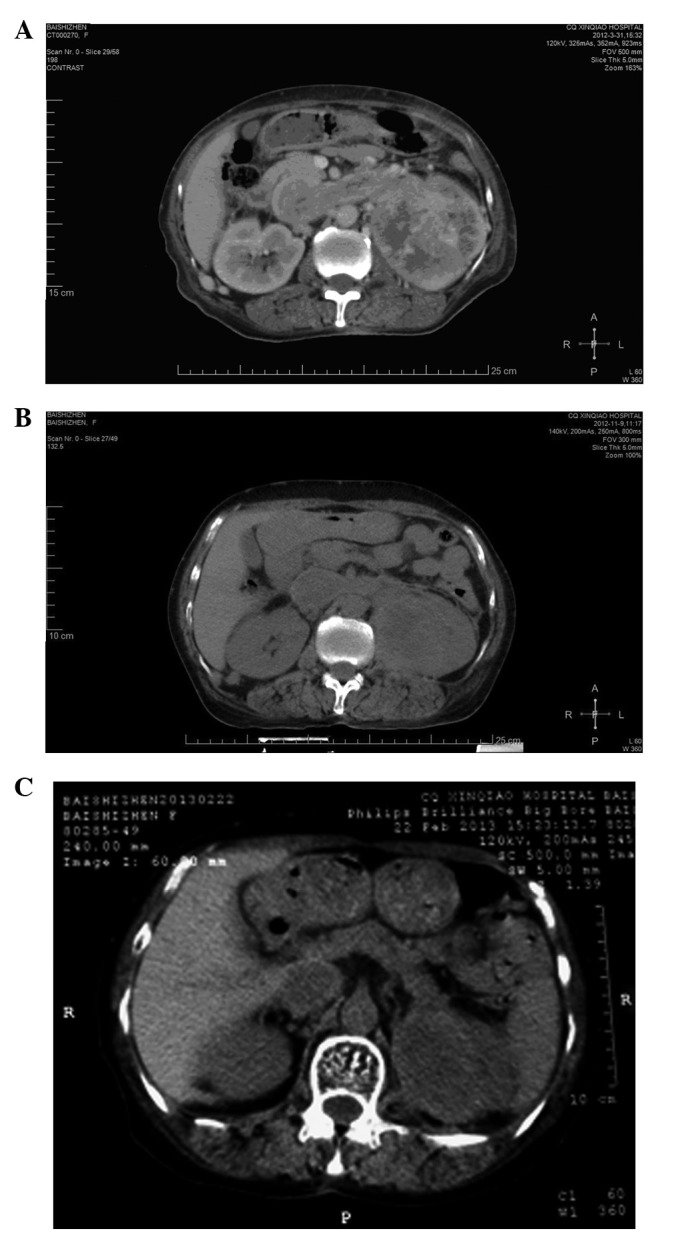
(A) Contrast enhanced computerized tomography (CT) scan shows a mass on the left kidney, and malignant thrombosis of the renal vein and inferior vena cava prior to radiation therapy. (B) The CT scan at the eight- and (C) 11-month follow-up.

On 16th March 2012, a 49-year-old female who experienced symptoms of exertional dyspnea and increased fatigue for 15 months presented at the Department of Cardiac Surgery, Xinqiao Hospital (Chongqing, China). The patient reported dyspnea, which developed following activity and sleeping in the supine position, which gradually worsened, without chest pain, heavy sweating and palpitations. The patient additionally reported poor sleep and a loss of appetite, although no marked weight loss. Two days prior to the the patient presenting at the hospital, the described symptoms worsened and the patient experienced bilateral edema in the lower limbs. Physical examination revealed cyanosis, an enlarged heart and a weakened cardiac sound. A transesophageal echocardiography demonstrated a right atrial mass originating in the inferior vena cava (IVC; size, 14×8 cm) that caused a tricuspid inflow obstruction and the abdomen ultrasonic examination demonstrated a mass in the lower right kidney. An emergency resection of the intra-atrial tumor thrombus was performed. Frozen sections obtained from the intra-atrial surgery revealed abundant necrosis with heterotypic cells. The intra-atrial tumor thrombus was tightly attached to the abdominal aorta, therefore, only a partial resection could be performed to relieve the tricuspid obstruction. Histological examination confirmed the diagnosis of a clear cell renal cell carcinoma, however, the patient’s symptoms had partially subsided following a month of recovery. The patient refused a right nephrectomy, and interleukin-2 and interferon-α therapy, as well as additional types of targeted therapy. The patient underwent intensity-modulated radiation therapy (56 Gy/24 fractions of one fraction per day, five days a week; from the IVC, where the mass initiated, to the iliac vein, which included the mass on the upper kidney). Following this, the patient refused further therapy. Six months following the radiation therapy, the symptoms had markedly improved, thus enhancing the patient’s quality of life; furthermore, the edema in the lower limbs had disappeared. On the 9^th^ November 2012 and the 22^nd^ February 2013, computed tomography (CT) indicated that the tumors on the kidney and IVC were stable (Fig. 1), however, additional metastatic lesions were identified in both lungs. Patient provided written informed consent.

## Discussion

SVC syndrome is difficult to treat, due to the complications associated with the pathology and the limited data that is available. The majority of reports for SVC are case studies and randomized trials are rare. In addition to treatment strategies to relieve symptoms, chemotherapy, surgical intervention and radiation therapy are commonly adopted methods of treatment; however, the control rate of the disease remains poor (2). According to previous reports, percutaneous placement of an intravascular stent, which offers rapid relief of the symptoms, is the most common intervention, due to its low level of complexity and low mortality rate (3). Surgical bypass grafting is an alternative approach that is more suitable in patients with no original malignancies or with malignancies that are not sensitive to treatment (4). A sternotomy or thoracotomy with extensive resection and reconstruction of the SVC are additional common therapeutic strategies (5).

More than 90% of SVC syndromes are attributable to a malignancy. Carcinoma of the bronchus is the most common cause, as well as lymphoma, germ-cell cancer, metastatic disease, thymoma and mesothelioma. The majority of SVC syndrome cases are caused by radiation-sensitive tumors (1), therefore, these particular patients may benefit from radiotherapy. It is generally considered that the treatment of clear cell renal cell carcinoma by radiation therapy is ineffective, however, metastatic lesions have been reported to be sensitive to and safe to treat using radiation therapy. Svedman *et al* (6) conducted a prospective phase II trial using extracranial stereotactic radiotherapy in primary and metastatic clear cell renal cell carcinomas. This treatment method was shown to achieve local control in 98% of treated lesions (6). Furthermore, a retrospective analysis demonstrated the feasibility of stereotactic radiosurgery (SRS)-based treatment of brain metastases originating from clear cell renal cell carcinoma in obtaining central nervous system control (7). These data indicated that radiotherapy may be a potential therapeutic strategy for clear cell renal cell carcinoma.

Sunitinib is a small molecule inhibitor that has been shown as an effective first-line therapeutic agent for metastatic clear cell renal cell carcinoma. Chiba *et al* (8) and Harshman *et al* (9) administered sunitinib (dose, 50 mg) as a neoadjuvant therapy in patients with clear cell renal cell carcinoma and a thrombus of the vena cava. The vena cava thrombus noticeably shrank following treatment (8). However, Cost *et al* (10) reported that targeted molecular therapy produced a minimal clinical effect on clear cell renal cell carcinoma thrombi. Consistently, a retrospective analysis of 14 patients by Bigot *et al* (11) showed that neoadjuvant targeted management therapy yielded a limited response on clear cell renal cell carcinoma patients with an IVC thrombus.

In conclusion, additional data are required to determine the efficacy of radiation therapy for the treatment of clear cell renal cell carcinoma-induced vena cava thrombi.

## References

[1] Wilson LD, Detterbeck FC, Yahalom J (2007). Clinical practice. Superior vena cava syndrome with malignant causes. N Engl J Med.

[2] Rowell NP, Gleeson FV (2001). Steroids, radiotherapy, chemotherapy and stents for superior vena caval obstruction in carcinoma of the bronchus. Cochrane Database Syst Rev.

[3] Urruticoechea A, Mesía R, Domínguez J (2004). Treatment of malignant superior vena cava syndrome by endovascular stent insertion. Experience on 52 patients with lung cancer. Lung Cancer.

[4] Kennedy DP, Palit TK (2010). Reconstruction of superior vena cava syndrome due to benign disease using superficial femoral vein. Ann Vasc Surg.

[5] Lequaglie C, Conti B, Brega-Massone PP, Giudice G (2003). The difficultapproach to neoplastic superior vena cava syndrome: surgical option. J Cardiovasc Surg (Torino).

[6] Svedman C, Sandström P, Pisa P (2006). A prospective Phase II trial of using extracranial stereotactic radiotherapy in primary and metastatic renal cell carcinoma. Acta Oncol.

[7] Samlowski WE, Majer M, Boucher KM (2008). Multidisciplinary treatment of brain metastases derived from clear cell renal cancer incorporating stereotactic radiosurgery. Cancer.

[8] Chiba H, Hirose T, Shimoda N, Kanagawa K (2012). A case of advanced renal cell carcinoma with inferior vena cava thrombus treated with sunitinib as neoadjuvant therapy. Nihon Hinyokika Gakkai Zasshi.

[9] Harshman LC, Srinivas S, Kamaya A, Chung BI (2009). Laparoscopicradical nephrectomy after shrinkage of a caval tumor thrombus with sunitinib. Nat Rev Urol.

[10] Cost NG, Delacroix SE, Sleeper JP (2011). The impact of targeted molecular therapies on the level of renal cell carcinoma vena caval tumor thrombus. Eur Urol.

[11] Bigot P, Fardoun T, Bernhard JC (2014). Neoadjuvant targeted molecular therapies in patients undergoing nephrectomy and inferior vena cava thrombectomy: is it useful?. World J Urol.

